# Association of brachial-ankle pulse wave velocity with cognitive impairment in peritoneal dialysis patients

**DOI:** 10.1080/0886022X.2021.1937221

**Published:** 2021-06-13

**Authors:** Chunyan Yi, Wenbo Zhang, Hongjian Ye, Haishan Wu, Xuan Huang, Jianxiong Lin, Xiao Yang

**Affiliations:** a Department of Nephrology,The First Affiliated Hospital, Sun Yat-sen University and Key Laboratory of Nephrology, Ministry of Health and Guangdong Province, Guangzhou, PR China; b Intensive Care Unit, The First Affiliated Hospital, Sun Yat-sen University, Guangzhou, PR China

**Keywords:** Peritoneal dialysis, cognitive function, brachial-ankle pulse wave velocity

## Abstract

**Background:**

The relationship between cognitive impairment (CI) and arterial stiffness in peritoneal dialysis (PD) patients has not been clearly clarified. The aim of this study was to examine the relationship between CI and arterial stiffness in PD patients.

**Methods:**

This cross-sectional study enrolled PD patients who performed a vascular profiler test at a single PD center in China between January 2014 and June 2016. The cognitive function was evaluated using the Montreal cognitive assessment (MoCA). A noninvasive vascular screening device was used to assess arterial stiffness relevant indicators.

**Results:**

A total of 643 PD patients with median age 45 (37–57.4) years and median duration of PD 27.8 (8.7–56.4) months were enrolled. The rate of CI was 49.9%. The mean brachial-ankle pulse wave velocity (baPWV) was 17.2 ± 5.6 m/s. Compared with normal cognitive function group, patients with CI had higher baPWV (18.6 ± 7.0 *vs.* 15.8 ± 3.2 m/s), systolic blood pressure (150.3 ± 21.5 *vs.* 144.2 ± 20.2 mmHg), and pulse pressure (59.7 ± 14.7 *vs.* 52.5 ± 11.6 mmHg), and lower ankle-brachial index (ABI, 1.12 ± 0.12 *vs.* 1.15 ± 0.09) (all *p*<.05). Compared with systolic blood pressure, pulse pressure, and ABI in receiver operating characteristic (ROC) analysis, baPWV had better performance in predicting CI (area under curve: 0.68, 95% confidence interval: 0.64–0.72). BaPWV was independently associated with MoCA score (B per SD, −0.42 [95% confidence interval, −0.71 to −0.12]; *p* = .006) and CI (OR per SD, 1.55 [95% confidence interval, 1.11–2.17]; *p* = .011) in PD patients after adjustment for confounders.

**Conclusions:**

Higher baPWV was independently associated with CI in PD patients.

## Introduction

The end-stage renal disease (ESRD) patients with peritoneal dialysis (PD) are prone to suffer from cognitive impairment (CI). The prevalence of CI in this population was reported as 3.3–86.8% [1–3]. The occurrence of CI may influence the ability of safe dialysate exchange and complex medication compliance [3]. Previous studies have shown that dialysis patients with CI have higher risk for PD-related peritonitis [4] and mortality [5]. However, the etiology of CI in this population has not been clearly elucidated. Some studies have demonstrated that older age, female, diabetes, low educational level, anemia, malnutrition, inflammation, and inadequate dialysis were risk factors for CI in dialysis patients [3,6–10]. But a few longitudinal studies showed inconsistent results [11–13]. Therefore, it is necessary to further explore the risk factors of CI in order to identify possible intervention measures and improve the prognosis of PD patients.

Arterial stiffness is a marker of functional and structural changes in arteries. Arterial stiffness has been appeared in the early stages of chronic kidney disease (CKD), and might be increased in ESRD patients due to deterioration of renal function, the deposit of abnormal product derived from proteins or lipids, the reduction of advanced glycation end products (AGEs), alteration of calcium and phosphate metabolism, low serum albumin levels, and high C-reactive protein serum levels [14,15]. As one of exact vascular risk factors, arterial stiffness has been demonstrated to be associated with CI in elderly population [16,17] and patients with chronic diseases such as hypertension [18–20] and diabetes mellitus [21,22]. However, there is a lack of related studies focuses on PD patients. So the purpose of this cross-sectional study was to explore the potential relationship between CI and arterial stiffness in PD patients.

## Materials and methods

### Study population and design

This cross-sectional study enrolled PD patients who performed a vascular profiler test at a single PD center in China between January 2014 and June 2016. All participants were 18 years or older and accepting continuous ambulatory PD treatment at least one month. The exclusion criteria were dementia, or transfer from permanent hemodialysis (HD), or failed renal transplantation, or lack of complete consent. The study protocol was approved by the hospital’s Human Ethics Committees. Written informed consents were obtained from all participants.

### Demographic and clinical data collection

Demographic data were obtained by medical history, which included age, gender, educational level, primary renal disease, duration of PD, and smoking status. Comorbidities were assessed by Charlson comorbidity index [23]. Clinical data were collected at enrollment, which included body mass index, hemoglobin, blood platelet count, high-sensitivity C-reactive protein, serum albumin, serum calcium, serum phosphorus, intact parathyroid hormone, total cholesterol, triglycerides, blood urea nitrogen, serum creatinine, residual renal function (measured glomerular filtration rate [mGFR]), and clearance index of urea (Kt/V).

### Measurements of cognitive function and arterial stiffness relevant indicators

Cognitive function of PD patients was assessed by the Montreal Cognitive Assessment (mandarin version) (MoCA) [24]. Two PD nurses were trained on the evaluation method of questionnaire before the study began. Then, the nurses used uniform language of instruction to assess the total score of MoCA of PD patients. The assessment content of MoCA included visuospatial and executive functions, attention, short-term memory, language, and orientation. The Cronbach’s alpha of the Chinese-Language MoCA was 0.78 for Mandarin population and 0.79 for Cantonese population [25]. The total score of MoCA is ranged from 0 to 30 points after adjustment for education. The previous study indicated that a cut off score of less than 26 points yielded a sensitivity of 90% and specificity of 78% for mild CI in older adults [24]. So CI was defined as the total score of MoCA less than 26 points in this study.

Within 1 year of the cognitive function assessment, the PD patients were tested for the arterial stiffness relevant indicators using a noninvasive vascular screening device (BP-203RPE III, Omron, Japan) when two liters of dialysate were injected into their abdominal cavity [26]. The validation and reproducibility of this device have been demonstrated by previous study [27]. Brachial-ankle pulse wave velocity (baPWV) is the velocity of the pulse wave that travels from the upper arm to the ankle joint. The formula of baPWV was as following: baPWV=(La-Lb)/Tba. The transmission distance from the brachium to ankle was calculated according to height of the patient. The path length from the suprasternal notch to the brachium (Lb) was obtained using the following equation: Lb = 0.2195 × height (cm)−2.0734. The path length from the suprasternal notch to the ankle (La) was obtained using the following equation: La = 0.8129 × height (cm)+12.328. Tba was the time interval between the initial increase in brachial and ankle waveforms. A higher PWV indicates poorer vessel wall elasticity and compliance, which contribute to increase in blood vessel stiffness [28]. Ankle-brachial index (ABI) was defined as ankle systolic blood pressure divided by brachial systolic blood pressure. As the blood flows from the central artery to the periphery, it encounters resistance and forms a reflection wave that builds up augmentation pressure at the end of contraction. Augmentation index (AI) refers to augmentation pressure divided by the height of entire systolic pressure wave (pulse pressure). Heart rate, systolic and diastolic blood pressure, and pulse pressure were also obtained by this device.

### Statistical analyses

All enrolled participants were divided into two groups according to whether the total score of MoCA was less than 26 points. Demographic, clinical data, and arterial stiffness relevant indicators between the two groups were compared. Normally distributed continuous variables were expressed as means and standard deviations. Continuous variables not normally distributed were expressed as medians and interquartile ranges. Categorical variables were expressed as frequencies and percentages. Independent-samples *t*-test, Mann–Whitney *U* test, and chi-squared test were used to test for differences in continuous or categoricalfactors between the two groups. Pearson correlation test was used to assess the correlations between total score of MoCA and arterial stiffness relevant indicators. Spearman rank correlation test was used to assess the correlations between CI and arterial stiffness relevant indicators. Receiver operating characteristic (ROC) analysis was used to calculate the sensitivity and specificity of arterial stiffness relevant indicators as diagnostic tool for CI. Multivariate linear regression analysis and binary logistic regression analysis were used to identify independent risk factors of cognitive function. According to presenting clinical relevance and avoiding multicollinearity, covariates with *p* < .05 in the univariate analysis were included in multivariate analysis. A two-tailed *p* < .05 was considered statistically significant. Statistical analysis was performed using SPSS version 16.0 (SPSS Inc., Chicago, IL).

## Results

### Demographic and clinical characteristics

**Figure 1.**
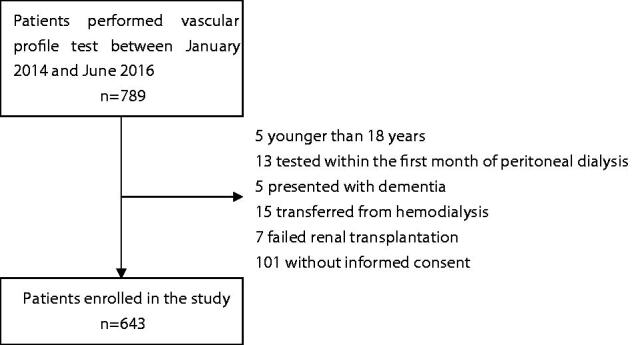
Flow chart.

Among 789 PD patients who performed vascular profile test, five patients were younger than 18 years, 13 patients were tested within the first month of PD, five patients presented dementia, 15 patients transferred from HD, seven failed renal transplantation, and 101 patients were not informed consent. Finally, 643 PD patients were enrolled in this study (Figure 1). The median age of participants was 45 (37–57.4) years, and the median duration of PD at enrollment was 27.8 (8.7–56.4) months. Among them, 42.1% of patients were female, and 16.3% with diabetes mellitus. The prevalence of CI is 49.9% (*n* = 321) in the study PD population. The mean total MoCA score was 24.1 ± 4.3 points in all patients, 20.8 ± 3.8 points in CI patients, and 27.5 ± 1.2 points in normal cognitive function patients, respectively. The mean baPWV was 17.2 ± 5.6 m/s in all patients, 18.6 ± 7.0 m/s in patients with CI, and 15.8 ± 3.2 m/s in patients with normal cognitive function, respectively.

### Comparison of clinical characteristics according to different cognitive function

Compared with normal cognitive function group, patients with CI had older age; longer duration of PD; higher proportion of female, diabetes mellitus and cardiovascular disease; lower educational level; higher Charlson comorbidity index score, body mass index, blood platelet count, high-sensitivity C-reactive protein, total cholesterol, triglyceride, baPWV, systolic blood pressure, and pulse pressure; lower level of serum albumin, serum calcium, serum creatinine, ABI, and total MoCA score (all *p* < .05) (Table 1).

**Table 1.** Comparison of demographic and clinical data.

Variables	Total (*n* = 643)	Cognitive impairment (*n* = 321)	Normal cognitive function (*n* = 322)	*p* Values
Age (years)	45 (37–57.4)	52.5 (43.2–62.7)	39.5 (33.6–47.4)	<.001
Female (*n*, %)	271 (42.1%)	158 (49.2%)	113 (35.1%)	<.001
Accepted below 12 years education (*n*, %)	289 (44.9%)	192 (59.8%)	97 (30.1%)	<.001
Primary renal disease (*n*, %)				<.001
Glomerulonephritis	445 (69.2%)	200 (62.3%)	245 (76.1%)	
Diabetic nephropathy	84 (13.1%)	67 (20.9%)	17 (5.3%)	
Renal vascular diseases	51 (7.9%)	29 (9.0%)	22 (6.8%)	
Other	63 (9.8%)	25 (7.8%)	38 (11.8%)	
Diabetes mellitus (*n*, %)	105 (16.3%)	84 (26.2%)	21 (6.5%)	<.001
Cardiovascular disease (*n*, %)	94 (14.6%)	66 (20.6%)	28 (8.7%)	<.001
Charlson comorbidity index (points)	3 (2–4)	3 (2–5)	2 (2–3)	<.001
Duration of peritoneal dialysis (months)	27.8 (8.7–56.4)	34.2 (9.7–59.6)	23 (7.5–50.8)	.009
Smoking (*n*, %)				.39
Never	492 (76.5%)	240 (76.9%)	252 (80.0%)	
Ever	66 (10.3%)	37 (11.9%)	29 (9.2%)	
Smoking	69 (10.7%)	35 (11.2%)	34 (10.8%)	
Body mass index (kg/m^2^)	22.4 ± 3.3	22.7 ± 3.4	22.1 ± 3.2	.03
Hemoglobin (g/L)	113.6 ± 18.9	112.9 ± 18.8	114.3 ± 19.0	.35
Blood platelet count (×10^9^/L)	238.0 ± 69.7	245.2 ± 72.2	230.8 ± 66.4	.009
High-sensitivity C-reactive protein (mg/L)	1.5 (0.6–4.0)	2.0 (0.8–5.3)	1.1 (0.5–3.2)	<.001
Serum albumin (g/L)	36.6 ± 4.4	35.7 ± 4.2	37.5 ± 4.4	<.001
Serum calcium (mmol/L)	2.3 ± 0.2	2.3 ± 0.2	2.3 ± 0.2	.01
Serum phosphorus (mmol/L)	1.6 (1.3–1.9)	1.5 (1.3–2.0)	1.6 (1.3–1.9)	.93
Intact parathyroid hormone (pg/mL)	290.7 (149.9–580.3)	279.7 (133.1–640.1)	293.6 (173.5–571.8)	.45
Total cholesterol (mmol/L)	5 (4.3–5.9)	5.2 (4.3–6.0)	4.9 (4.2–5.6)	.006
Triglycerides (mmol/L)	1.5 (1.1–2.1)	1.6 (1.1–2.2)	1.4 (1.1–2.1)	.04
Blood urea nitrogen (mmol/L)	17.5 (14.1–21.5)	17.7 (14.1–22.4)	17.3 (14.2–20.5)	.37
Serum creatinine (umol/L)	972 (751–1215)	901(704–1152.5)	1027.5 (795–1262.5)	<.001
Residual renal function (mL/min/1.73 m^2^)	1.3 (0.04–3.3)	1.2 (0.02–3.4)	1.5 (0.07–3.1)	.77
Clearance index of urea	2.2 (1.9–2.6)	2.3 (1.9–2.7)	2.2 (1.9–2.6)	.09
Brachial pulse wave velocity (m/s)	17.2 ± 5.6	18.6 ± 7.0	15.8 ± 3.2	<.001
Heart rate (bpm)	78.2 ± 19.1	79 ± 19.2	77.2 ± 19	.22
Systolic blood pressure (mmHg)	147.2 ± 21	150.3 ± 21.5	144.2 ± 20.2	<.001
Diastolic blood pressure (mmHg)	91.2 ± 13.0	90.6 ± 12.8	91.7 ± 13.1	.29
Pulse pressure (mmHg)	56.1 ± 13.7	59.7 ± 14.7	52.5 ± 11.6	<.001
Ankle-brachial index	1.14 ± 0.11	1.12 ± 0.12	1.15 ± 0.09	<.001
Augmentation index	11.7 (−0.5–24.9)	11.9 (1.2–24.6)	11.4 (−2.0–24.9)	.22
Score of Montreal cognitive assessment (points)	24.1 ± 4.3	20.8 ± 3.8	27.5 ± 1.2	<.001

#### Correlation between arterial stiffness relevant indicators and cognitive function

**Figure 2.**
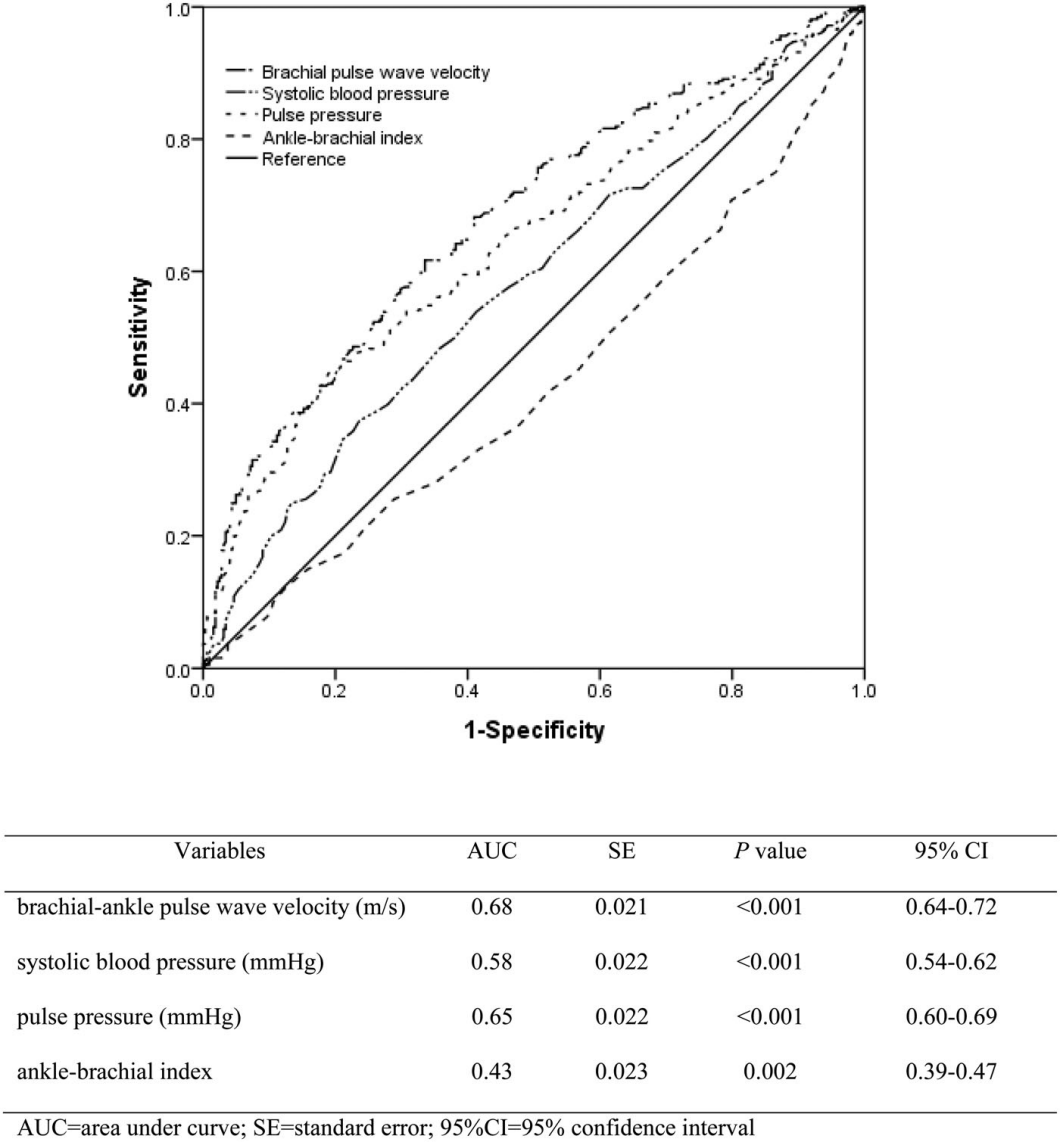
Performance of different measurement of arterial stiffness in predicting cognitive impairment in the receiver operating characteristic analysis.

Pearson correlation analysis showed that total MoCA score was correlated with baPWV, heart rate, systolic blood pressure, pulse pressure, and ABI (all *p* < .05) (Table 2). Spearman’s correlation analysis showed that baPWV, heart rate, systolic blood pressure, and pulse pressure were positively correlated with CI, while ABI was negatively correlated with CI (all *p* < .05) (Table 2). The ROC analysis was used for evaluating the performance of different arterial stiffness relevant indicators in predicting CI. Figure 2 showed that baPWV had a best discrimination and calibration for predicting CI compared with systolic blood pressure, pulse pressure, and ABI, with a higher area under the curve of 0.68 (95% confidence interval: 0.64–0.72).

**Table 2.** Correlation between Arterial stiffness relevant indicators and cognitive function.

Variables	Total Score of MoCA		Cognitive impairment	
	*r*	*p* Values	*r'*	*p* Values
Brachial pulse wave velocity (m/s)	−0.35	<.001	0.32	<.001
Heart rate (bpm)	−0.08	.03	0.08	.05
Systolic blood pressure (mmHg)	−0.14	<.001	0.14	<.001
Diastolic blood pressure (mmHg)	0.07	.09	−0.05	.19
Pulse pressure (mmHg)	−0.26	<.001	0.25	<.001
Ankle-brachial index	0.16	<.001	−0.12	.002
Augmentation index	−0.07	.09	0.05	.23

MoCA: Montreal Cognitive Assessment; *r*: Pearson correlation coefficient; *r*': Spearman’s correlation coefficient.

#### Association of baPWV with cognitive function

Univariate linear regression analysis showed that older age, female, lower educational level, diabetes mellitus, duration of PD, blood platelet count, high-sensitivity C-reactive protein, serum albumin, total cholesterol, serum creatinine and baPWV were associated with total score of MoCA in PD patients (all *p* < .05) (Table 3). These factors and smoking status were included into multivariate linear regression analysis. The results showed that baPWV was independently associated with total score of MoCA in PD patients after adjustment for the underlying cofounders (B per SD, −0.42 [95% confidence interval, −0.71 to −0.12]; *p* = .006) (Table 3).

**Table 3.** Associations between brachial pulse wave velocity and score of Montreal cognitive assessment by linear regression analysis.

Variables	Univariate linear regression			Multivariate linear regression		
	B	95%CI for B	*p* Values	B	95%CI for B	*p* Values
Age (per 1 year)	−0.17	−0.19 to −0.15	<.001	−0.12	−0.14 to −0.09	<.001
Female (yes)	−1.59	−2.26 to −0.91	<.001	−0.94	−1.59 to −0.28	.005
Accepted below 12 years education (yes)	−3.16	−3.79 to −2.53	<.001	−2.07	−2.63 to −1.51	<.001
Diabetes mellitus (yes)	−3.49	−4.36 to −2.62	<.001	−1.47	−2.27 to −0.67	<.001
Duration of peritoneal dialysis (per 1 month)	−0.01	−0.02 to −0.001	.035	−0.001	−0.01 to −0.01	.778
Smoking (yes)	−0.09	−0.61–0.43	.731	−0.01	−0.47–0.44	.955
Blood platelet count (per 1 × 10^9^/L)	−0.01	−0.01 to −0.002	.006	−0.002	−0.01–0.002	.367
High-sensitivity C-reactive protein (per 1 mg/L)	−0.06	−0.10 to −0.03	.001	−0.01	−0.05–0.02	.457
Serum albumin (per 1 g/L)	0.21	0.13–0.28	<.001	0.05	−0.02–0.11	.166
Total cholesterol (per 1 mmol/L)	−0.62	−0.90 to −0.34	<.001	−0.24	−0.48–0.01	.062
Serum creatinine (per 1 umol/L)	0.003	0.002–0.004	<.001	0	−0.001–0.001	.372
Brachial pulse wave velocity (per 5.6 m/s)	−1.30	−1.62 to −0.98	<.001	−0.42	−0.71–−0.12	.006

B, unstandardized coefficients; 95% CI；95% confidence interval.

*Note*: Odds ratio and 95% confidence interval of brachial pulse wave velocity were calculated for a 1 standard deviation increase in linear regression analysis.

Univariate binary logistic regression analysis showed that older age, female, lower educational level, diabetes mellitus, duration of dialysis, body mass index, blood platelet count, high-sensitivity C-reactive protein, serum albumin, serum calcium, total cholesterol, serum creatinine, baPWV, systolic blood pressure, pulse pressure, and ABI were associated with CI in PD patients (all *p* < .05) (Table 4). These factors and smoking status were included into multivariate binary logistic regression analysis. And the results showed that baPWV, systolic blood pressure, and pulse pressure was independently associated with CI in PD patients after adjustment for the underlying cofounders (all *p* < .05) (Table 5).

**Table 4.** Univariate binary logistic regression analysis for the influence factors of cognitive impairment in peritoneal dialysis patients.

Variables	OR	95% CI	*p* Values
Age (per 1 year)	1.08	1.06–1.09	<.001
Female (yes)	1.79	1.31–2.46	<.001
Accepted below 12 years education (yes)	3.45	2.49–4.78	<.001
Diabetes mellitus (yes)	5.08	3.06–8.44	<.001
Duration of peritoneal dialysis (per 1 month)	1.01	1.001–1.01	.014
Smoking (yes)	1.08	0.86–1.37	.509
Body mass index (per 1 kg/m^2^)	1.05	1.01–1.11	.032
Blood platelet count (per 1× 10^9^/L)	1.00	1.00–1.01	.010
High-sensitivity C-reactive protein (per 1 mg/L)	1.03	1.01–1.05	.014
Serum albumin (per 1 g/L)	0.91	0.88–0.95	<.001
Serum calcium (per 1 mmol/L)	0.37	0.17–0.81	.013
Total cholesterol (per 1 mmol/L)	1.22	1.07–1.40	.003
Serum creatinine (per 1 umol/L)	0.99	0.99–1.00	<.001
Brachial pulse wave velocity (per 5.6 m/s)	2.84	2.12–3.80	<.001
Systolic blood pressure (per 21 mmHg)	1.35	1.15–1.58	<.001
Pulse pressure (per 13.7 mmHg)	1.76	1.49–2.09	<.001
Ankle-brachial index (per 0.11)	0.76	0.64–0.90	.002

OR: odds ratio; 95% CI: 95% confidence interval

*Note*: For arterial stiffness relevant indicators, odds ratios and 95% confidence intervals were calculated for a 1 standard deviation increase in univariate binary logistic regression analysis.

**Table 5.** Associations between arterial stiffness relevant indicators and cognitive impairment by binary logistic regression analysis.

Model	Model 1		Model 2		Model 3	
	OR (95% CI)	*p* Values	OR (95% CI)	*p* Values	OR (95% CI)	*p* Values
Brachial pulse wave velocity (per 5.6 m/s)	2.84 (2.12–3.80)	<.001	1.65 (1.18–2.32)	.004	1.55 (1.11–2.17)	.011
Systolic blood pressure (per 21 mmHg)	1.35 (1.15–1.58)	<.001	1.32 (1.10–1.59)	.003	1.33 (1.09–1.62)	.006
Pulse pressure (per 13.7 mmHg)	1.76 (1.49–2.09)	<.001	1.33 (1.08–1.63)	.006	1.34 (1.08–1.67)	.009
Ankle-brachial index (per 0.11)	0.76 (0.64–0.90)	.002	1.03 (0.84–1.27)	.771	1.03 (0.83–1.28)	.788

OR: odds ratio; 95% CI: 95% confidence interval

*Note*: For arterial stiffness relevant indicators, odds ratios and 95% confidence intervals were calculated for a 1 standard deviation increase in binary logistic regression analysis.

## Discussion

This cross-sectional study demonstrated that the prevalence of CI in the study PD population was 49.9% assessed by the MoCA. The mean baPWV was 17.2 ± 5.6 m/s. Compared with systolic blood pressure, pulse pressure, and ABI, baPWV had better performance in predicting CI. After adjustment for the underlying confounders, it was found that higher baPWV was independently associated with CI in PD patients.

In this study, the prevalence of CI in PD patients was 49.9% through MoCA test. A systematic review and meta analysis showed that the prevalence of CI in PD patients ranged from 3.3% to 74.5% and the overall pooled prevalence of CI was 28.7%, which analyzed the relevant data from 1736 PD patients in eight studies [29]. The different results of these studies may be due to different cognitive function assessment tools and different demographic characteristics of patients. Xu et al. [30] applied the modified mini–mental state examination, trail making tests A and B, subtests of Repeatable Battery for the Assessment of Neuropsychological Status to assessed the cognitive function of 476 PD patients from 5 PD units in China, and found that the prevalence of CI was 28.4% in PD patients with the mean age 51.9 ± 14.3 years and the proportion of diabetes mellitus 23.7%. Zheng et al. [2] enrolled 72 PD patients with the mean age 56.2 ± 16.0 years and the proportion of diabetes mellitus 31.9%, and found that 25% and 86.8% of PD patients could be diagnosed as CI, according to the MMSE and MoCA test, respectively. But overall, these findings suggested that CI was common in patients with PD.

Our results showed that the mean baPWV in PD patients was higher than that in healthy adults, which was similar to other literature reports [31,32]. The relationship between arterial stiffness and CKD is complex. CKD is associated with accelerated vascular aging. Activation of renin–angiotensin system, aortic inflammation, and vascular metalloproteinase activity lead to changes in the extracellular matrix and endothelial dysfunction, which thereby increase arterial stiffness [33]. Through both a reduction in baroreflex sensitivity and the passive effect of loss of arterial wall elastic properties, arterial stiffness increases the variability of blood pressure, which in turn might contribute to the development and progression of kidney damage [34]. In the process from CKD to ESRD, premature aging of the vascular system leads to extreme increases in arterial stiffness [35]. Hypertension, disorder of lipid metabolism and calcium and phosphate metabolism, accumulation of uremic toxins such as AGEs, and chronic inflammation associated with peritoneal fluid biocompatibility are common in PD patients. These risk factors also contribute to the progression of arterial stiffness [14].

Through noninvasive vascular screening device, arterial stiffness can be assessed using various indicators such as PWV, ABI, AI, cardio-ankle vascular index, etc. Carotid-femoral PWV (cfPWV) is considered the gold standard method for assessing aortic stiffness [36]. However, measuring cfPWV with tonometer or doppler requires specialized training and exposure to the groin area, and is thought to be not easy to operate in clinical practice [37]. Sharing the same theoretical background with cfPWV, baPWV is easier to use clinically because it just requires wrapping of a pressure cuff in each of the exposed extremities [27]. Although baPWV reflects the stiffness of small arteries (especially the muscular arteries of the arms and legs) and does not fully reflect the stiffness of the central artery [38], it is closely correlated with the directly measured aortic PWV and cfPWV [39]. As the ROC curves of this study showed, baPWV had higher area under the curve for predicting CI compared with systolic blood pressure, pulse pressure, and ABI.

Some previous studies have demonstrated the association between arterial stiffness and CI with inconsistent results [7,40,41]. However, this association has not been reported in PD patients. Our results found that higher baPWV was independently associated with CI in PD patients. Consistent with our results, Angermann et al. found that higher baPWV was independently associated with CI in 201 HD patients by MoCA test [7]. Another study also showed the positive association of cfPWV with CI among HD patients [40]. But a resent prospective study did not show significant associations between cfPWV and cognitive function in HD patients, mainly due to the relatively younger age of study population [41]. The possible mechanisms of the association between arterial stiffness and CI were as following. First, greater arterial stiffness can lead to microcirculatory damage of the brain through increasing pulsatile pressure and flow load [42]. Second, high pulse pressure may lead to structural changes in the cerebral vessels that may interfere with the transport of important nutrients to the brain and the removal of toxic byproducts from the brain [43]. Finally, brain imaging studies have found arterial stiffness was associated with cerebral microvascular disease and changes of cerebral white matter lesions, which in turn were associated with CI [44,45].

One of strengths of this study was that it was the first to reveal the association between arterial stiffness and CI in PD patients. Another strength was large sample of study to reduce selection bias. However, there were some limitations of this study. First, the neuroimaging was not performed for participants, so it was possible that the rate of CI might be underestimated. Second, inter-rater and intra-rater reliability of MoCA were not measured in this study, which might cause bias. Finally, this study could not make the causal inferences because of a cross-sectional nature. Longitudinal studies are needed to determine if higher baPWV predicts cognitive decline in PD patients.

## Conclusions

In this cohort of PD patients, we indicated that higher baPWV was independently associated with CI of PD patients. Further prospective studies need to confirm the association of baPWV and cognitive decline in PD patients.
